# Translational outcomes in a full gene deletion of ubiquitin protein ligase E3A rat model of Angelman syndrome

**DOI:** 10.1038/s41398-020-0720-2

**Published:** 2020-01-27

**Authors:** E. L. Berg, M. C. Pride, S. P. Petkova, R. D. Lee, N. A. Copping, Y. Shen, A. Adhikari, T. A. Fenton, L. R. Pedersen, L. S. Noakes, B. J. Nieman, J. P. Lerch, S. Harris, H. A. Born, M. M. Peters, P. Deng, D. L. Cameron, K. D. Fink, U. Beitnere, H. O’Geen, A. E. Anderson, S. V. Dindot, K. R. Nash, E. J. Weeber, M. Wöhr, J. Ellegood, D. J. Segal, J. L. Silverman

**Affiliations:** 1 MIND Institute and Department of Psychiatry and Behavioral Sciences, University of California Davis School of Medicine, Sacramento, CA USA; 2 Mouse Imaging Centre, Toronto Centre for Phenogenomics, The Hospital for Sick Children, Toronto, ON Canada; 3 Wellcome Centre for Integrative Neuroimaging, The University of Oxford, Oxford, UK; 4 Department of Pediatrics and Neurology, Baylor College of Medicine, Houston, TX USA; 5 Department of Molecular Pharmacology and Physiology, University of South Florida, Tampa, FL USA; 6 Stem Cell Program, Institute for Regenerative Cures, and Department of Neurology, University of California Davis School of Medicine, Sacramento, CA USA; 7 MIND Institute, Genome Center, and Department of Biochemistry and Molecular Medicine, University of California Davis, Davis, CA USA; 8 Department of Veterinary Pathobiology, College of Veterinary Medicine and Biomedical Sciences, Texas A&M University, College Station, TX USA; 9 Behavioral Neuroscience, Experimental and Biological Psychology, Philipps-University of Marburg, Marburg, Germany

**Keywords:** Autism spectrum disorders, Genetics

## Abstract

Angelman syndrome (AS) is a rare neurodevelopmental disorder characterized by developmental delay, impaired communication, motor deficits and ataxia, intellectual disabilities, microcephaly, and seizures. The genetic cause of AS is the loss of expression of *UBE3A* (ubiquitin protein ligase E6-AP) in the brain, typically due to a deletion of the maternal 15q11-q13 region. Previous studies have been performed using a mouse model with a deletion of a single exon of *Ube3a*. Since three splice variants of Ube3a exist, this has led to a lack of consistent reports and the theory that perhaps not all mouse studies were assessing the effects of an absence of all functional UBE3A. Herein, we report the generation and functional characterization of a novel model of Angelman syndrome by deleting the entire *Ube3a* gene in the rat. We validated that this resulted in the first comprehensive gene deletion rodent model. Ultrasonic vocalizations from newborn *Ube3a*^*m−/p+*^ were reduced in the maternal inherited deletion group with no observable change in the *Ube3a*^*m+/p−*^ paternal transmission cohort. We also discovered *Ube3a*^*m−/p+*^ exhibited delayed reflex development, motor deficits in rearing and fine motor skills, aberrant social communication, and impaired touchscreen learning and memory in young adults. These behavioral deficits were large in effect size and easily apparent in the larger rodent species. Low social communication was detected using a playback task that is unique to rats. Structural imaging illustrated decreased brain volume in *Ube3a*^*m−/p+*^ and a variety of intriguing neuroanatomical phenotypes while *Ube3a*^*m+/p−*^ did not exhibit altered neuroanatomy. Our report identifies, for the first time, unique AS relevant functional phenotypes and anatomical markers as preclinical outcomes to test various strategies for gene and molecular therapies in AS.

## Introduction

Angelman syndrome (AS) is a rare neurodevelopmental disorder characterized by developmental delay, impaired communication skills, ataxia, motor and balance deficits, poor attention, intellectual disabilities, microcephaly, and seizures^1–3^. AS is caused by loss-of-expression or loss-of-function of the maternally inherited allele of the Ubiquitin protein ligase E3A (*UBE3A* E6-AP*)*, which typically arises through a de novo deletion in the maternal 15q11-q13 region^4–6^. Owing to genomic imprinting, the paternal allele is silenced in neurons of the central nervous system (CNS). Angelman syndrome is thus caused by loss of UBE3A in neurons of the CNS^7^.

The Foundation for Angelman Syndrome Therapeutics (FAST) funded the generation of a genetic rat model of AS via a 90-kb deletion on chromosome 1, which includes the entire *Ube3a* gene. This has opened new possible avenues of research into the neurobiological and behavioral effects of loss of all isoforms of UBE3A and, crucially, the development of novel therapeutics in the near future, including gene replacement therapies. This unique rat model of AS also provides opportunities to investigate complex AS relevant behaviors that have been difficult to capture with highsignal sensitivity, rigor, and reproducibility in mice, such as behaviors across developmental time points, juvenile acoustic social communication, and cognitive dysfunction.

Well-validated tools for behavioral and functional outcomes for neurodevelopmental disorders have been well standardized^8^, but sophisticated social communication, translationally relevant learning and memory, and other AS-symptom domains are less developed in mice^9^. One prominent example is the less complex acoustic communication system in the mouse. Rats emit uniquely detectable ultrasonic vocalizations (USV) that serve as situation-dependent evolved signals and that accomplish important communicative functions as alarm or social contact calls^10–12^. Another advantage of a rat model is the ability to utilize advanced cognitive tests for measuring learning and memory. Examining cognitive functions in an evolutionarily advanced species^13,14^ through the use of behavioral tests highly relevant to clinical diagnostic assays may improve translational predictability.

The present experiments aimed to take advantage of the first generated rat model of a complete *Ube3a* deletion and define behavioral and anatomical phenotypes by utilizing our comprehensive battery of standardized and innovative outcome measures to identify functional outcomes relevant to AS. Using sophisticated and nuanced behavioral readouts of isolation-induced USV, juvenile social communication via USV playback, computerized touchscreen learning and memory, and magnetic resonance imaging (MRI), we evaluated various aspects of social communication, cognition, and behavior during development in the AS rat model.

## Results

### Model generation

The *Ube3a*^*m−/p+*^ rat line (background Sprague-Dawley) was originally designed by the Segal laboratory using the CRISPR-Cas9 system, generated by Transposagen (Fig. S1). Two genomic RNAs (gRNAs) were designed to target the 5ʹ-end of the *Ube3a* gene (upstream of the *Ube3a* coding sequence) and two gRNAs target sequences downstream of *Ube3a*. gRNA pairs were used on each end of the deletion to maximize the probability of a complete deletion of the 90-kb region encompassing the *Ube3a* gene. 5′ CRISPR-1 Target site GGCCCTGCAGAGATGCAATC, 5′CRISPR-2 Target site GGAGCCCTCCGCCGGCA, 3′CRISPR-1 Target site TACCCTTCCCAGGCCCC, and 3′CRISPR-2 Target site GCATTTCTAGTACATCATCC. In addition, a bridging DNA fragment was constructed with 600-bp homology to the sequence upstream and homology to 1-kb downstream of the deletion. The Rnor_6.0 genome build coordinates of homology arms are 116587209–116587779 and 116678173–116679214, respectively. CRISPR/gRNA complexes were co-injected with the “bridging construct” into fertilized Sprague-Dawley rat embryos and inserted into a surrogate. Founders were screened for deletion of the entire 90-kb region and germline transmission was confirmed using genotyping primers (Ube3aDel-F: 5′-ACCTAGCCCAAAGCCATCTC-3′ and Ube3aDel-R: GGGAACAGCAAAAGACATGG-3′). Junction of deletion of the entire Ube3a gene (~90 kb) was confirmed by Sanger sequencing (Fig. S1). Deletion was further validated by Western blotting (Fig. S2). For transparency, we do have knowledge via foundation collaboration and conference presentations that another laboratory has access to these novel AS rats and is working on adult characterization and long-term potentiation (personal communication).

### Reduced isolation-induced pup ultrasonic vocalizations (USV) and delayed neonatal reflex development in *Ube3a*^*m−/p+*^ pups

**Fig. 1:**
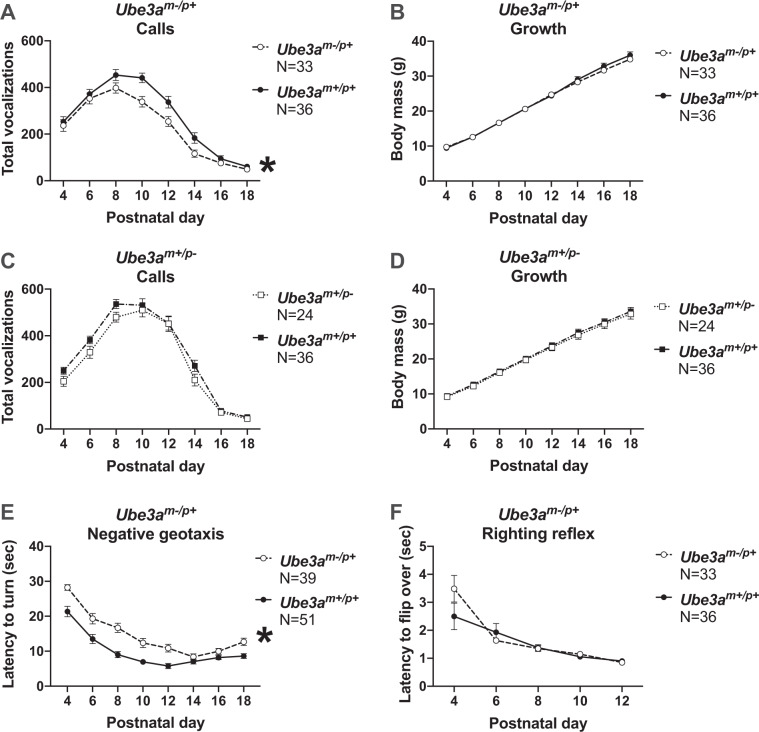
Reduced isolation-induced pup ultrasonic vocalizations and delayed neonatal reflex development in *Ube3a*^*m−/p+*^ pups. **a** *Ube3a*^*m−/p+*^ pups emitted significantly fewer USV across early development compared to wildtypes, and **b** demonstrated normal weight gain. **c** *Ube3a*^*m+/p−*^ pups emitted normal numbers of USV across early development, and **d** also demonstrated normal weight gain. **e** Compared to wildtype littermates, *Ube3a*^*m−/p+*^ pups were significantly slower in the negative geotaxis test but **f** had normal latencies to flip over in the test of righting reflex. All analyses include males and females. Mean +/− S.E.M. is depicted. **a**–**f**: *p *<* 0.05, repeated measures ANOVA, main effect of genotype.

Pup ultrasonic vocalizations (USV) of infant rats and mice measure an early communicative behavior between pups and mother. Isolation-induced USV were collected for 3 min as social communication signals in rat pups on postnatal day (PND) 4, 6, 8, 10, 12, 14, 16, and 18, as previously described^15^. *Ube3a*^*m−/p+*^ pups emitted significantly fewer USV across early development compared to wildtype *Ube3a*^*m+/p+*^ littermate controls (Fig. 1a*F*_(1, 67)_ = 10.80, *p* < 0.002). Holm-Sidak corrected posthoc analysis for multiple comparisons highlights PND 10 and PND 12 as reduced in the *Ube3a*^*m−/p+*^ compared to *Ube3a*^*m+/p+*^ littermates (PND 10: *p* = 0.0023; PND 12: *p* = 0.022) with a trend on PND 14 (*p* = 0.091). *Ube3a*^*m−/p+*^ pups also emitted significantly fewer USV on PND 8 in the Baylor laboratory compared to wildtype *Ube3a*^*m+/p+*^ littermate controls, independently reproducing our results (Fig. S3*t*_(1, 23)_ = 2.991, *p* < 0.007). Body weight and temperature were also collected to assure that the reduced USV were not the result of being physically smaller as body weight is known to alter pup USV emission^16,17^. Weight did not differ between genotypes (Fig. 1b*F*_(1, 67)_ = 0.154, *p* > 0.05) indicating typical growth and ability to thrive. As expected, pups with paternal inheritance of the deletion (*Ube3a*^*m+/p−*^) did not have reductions in USV emissions (Fig. 1c*F*_(1, 58)_ *=* 3.555, *p* > 0.05) or body weight across early development (Fig. 1d*F*_(1, 58)_ *=* 0.140, *p* > 0.05), compared to wildtype *Ube3a*^*m+/p+*^ littermate controls. Body temperature did not differ between genotypes (*Ube3a*^*m−/p+*^ versus *Ube3a*^*m+/p+*^*: F*_(1, 67)_ = 3.859, *p* > 0.05 and *Ube3a*^*m+/p−*^ versus *Ube3a*^*m+/p+*^*: F*_(1, 58)_ = 0.038, *p* > 0.05). Supplementary Tables S1 and S2 show mostly typical early physical development and neurological reflexes in various parameters in *Ube3a*^*m−/p+*^ versus *Ube3a*^*m+/p+*^ littermates. Interestingly, longer latencies to navigate upright in negative geotaxis, a simple metric for motoric, postural, and proprioceptive processes that underlie the ability of infant rodents to navigate on an inclined plane, was robustly delayed in the *Ube3a*^*m−/p+*^ versus *Ube3a*^*m+/p+*^ (Fig. 1e*F*_(1, 88)_ **=** 37.22, *p* < 0.0001). Longer latencies were observed for 6 of the 8 days tested (Bonferroni-corrected *p* < 0.05): PND 4, 6, 8, 10, 12, and 18. Yet, similar latencies to flip over 180 degrees from supine to prone in the test of righting reflex were observed (Fig. 1f*F*_(1, 71)_ **=** 0.651, *p* = 0.422).

### Reduced vertical activity, poor rotarod performance, and long latencies to remove adhesive illustrate poor gross and fine motor abilities in *Ube3a*^*m−/p+*^ rats

**Fig. 2:**
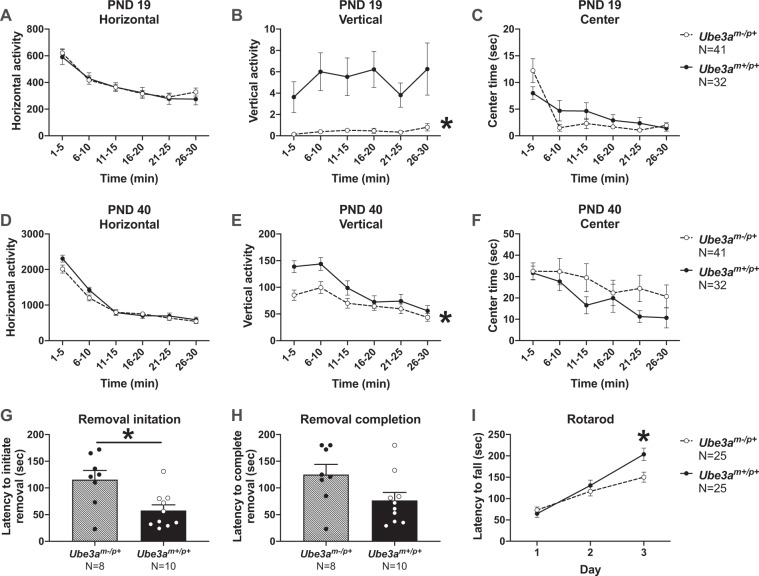
Reduced vertical activity and motor learning deficit in *Ube3a*^*m−/p+*^ rats. **a** At PND 19, *Ube3a*^*m−/p+*^ juvenile rats exhibited normal horizontal activity but **b** had significantly reduced vertical activity compared to wildtypes. **c** Center time did not differ between groups. **d** At PND 40, *Ube3a*^*m−/p+*^ rats again exhibited normal horizontal activity, **e** significantly lower vertical activity, and **f** normal center time. Note: the difference in axis labels shows how much more active PND 40 rats are over PND 19. **g** In the adhesive removal task, *Ube3a*^*m−/p+*^ rats had significantly higher latencies to initiate removal of the adhesive. **h** Latency to completely remove the adhesive did not differ between groups but trended longer (*p* = 0.059) in the *Ube3a*^*m−/p+*^ rats, suggesting fine motor skill deficits of the fore paws. **i** *Ube3a*^*m−/p+*^ rats had significantly lower latencies to fall of the rotarod on day 3 compared to wildtypes. Analyses include both males and females. Mean +/− S.E.M. is depicted. **a**–**f**: *p *<* 0.05, repeated measures ANOVA, main effect of genotype. **g**–**h**: *p < 0.05, Student’s *t*-test. **i**: *p < 0.05, repeated measures ANOVA, Holm-Sidak’s multiple comparisons test.

Motor abilities were tested in an open field assay, assessing cm^2^ of horizontal and vertical movements using beam breaks and time spent in the center of the arena. At PND 19, *Ube3a*^*m−/p+*^ juvenile rats exhibited normal horizontal activity (Fig. 2a*F*_(1, 71)_ = 0.1866, *p* > 0.05) yet significantly reduced vertical activity (Fig. 2b*F*_(1, 71)_ = 18.55, *p* < 0.0001) compared to wildtype *Ube3a*^*m+/p+*^ littermate controls for every 5-min time bin across the 30-min task. No genotype differences were detected in center time measures at either the early or later developmental time points (Fig. 2c PND 19 *F*_(1, 71)_ = 0.1866, *p* > 0.05 and Fig. 2f PND 40 *F*_(1, 71)_ = 1.397, *p* > 0.05). Young-adult (PND 40) *Ube3a*^*m−/p+*^ rats exhibited normal horizontal activity (Fig. 2d*F*_(1, 71)_ = 1.266, *p* > 0.05) yet significantly reduced vertical activity (Fig. 2e*F*_(1, 71)_ = 5.882, *p* < 0.02) compared to wildtype *Ube3a*^*m+/p+*^ littermate controls. In the adhesive removal task, *Ube3a*^*m−/p+*^ rats were significantly slower to initiate removal of the sticker (Fig. 2g*t* = 2.986, df = 16, *p* < 0.009), and trended slower to complete adhesive removal (Fig. 2h*t* = 2.032, df = 16, *p* = 0.059), suggesting fine motor skill deficits of the fore paws; the *Ube3a*^*m−/p+*^ rats were 48.63 +/− 23.93 sec slower to finish removing the adhesive. *Ube3a*^*m*−/*p*+^ rats had normal rotarod performance on the first 2 days of testing but were significantly faster to fall off the rotarod on the third day compared to wildtypes (Fig. 2i time × genotype interaction: *F*_(2, 96)_ = 7.339, *p* = 0.001; day 3 Holm-Sidak *p* = 0.0197), highlighting a motor learning deficit.

### Reduced exploration of pro-social 50-kHz USV in *Ube3a*^*m−/p+*^ juvenile rats

**Fig. 3:**
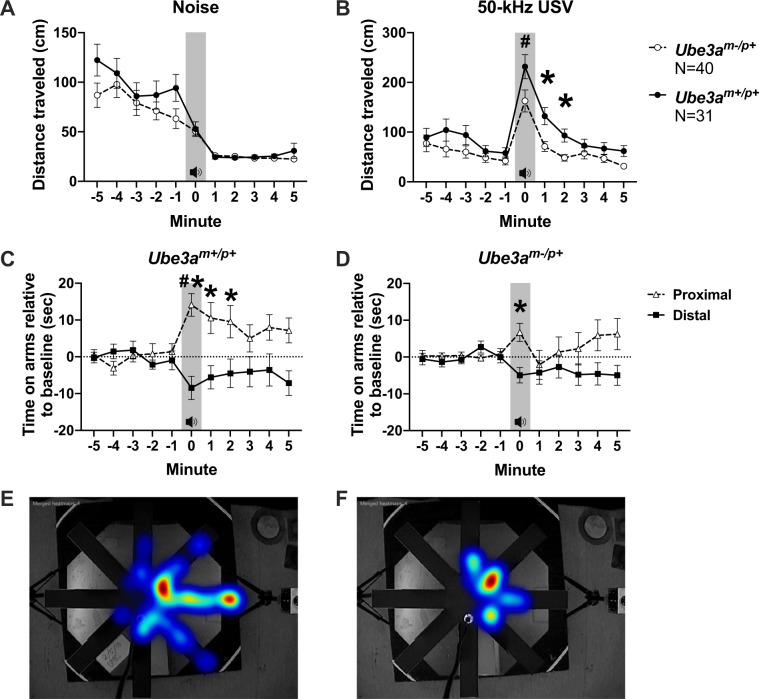
Atypical social communication in *Ube3a*^*m−/p+*^ juveniles using 50-kHz pro-social ultrasonic vocalizations. **a** Distance traveled in response to the white noise control stimulus (gray zone) did not differ between groups: both exhibited behavioral inhibition (i.e., a reduction in motion following the noise control). **b** Distance traveled increased in response to playback of 50-kHz USV (gray zone) in *Ube3a*^*m−/p+*^ and *Ube3a*^*m+/p+*^ rats. Interestingly, the duration of the response was shorter in *Ube3a*^*m−/p+*^ compared to that of wildtypes. **c** In the radial maze used, time spent in the arms proximal to the active ultrasonic speaker versus time spent in the distal arms indicate social interest, preference and social engagement. *Ube3a*^*m+/p+*^ rats spent significantly longer time on the arms proximal to the speaker emitting the 50-kHz USV upon playback and for several minutes afterwards showing a strong, sustained social response. **d** *Ube3a*^*m−/p+*^ subjects failed to show a strong, sustained response to hearing the USV and only spent significantly more time on the proximal arms during the initial time period the audio cue was “on” (gray zone). **e**, **f** Representative heat maps of the distance and direction traveled in response to the 50-kHz USV. Analyses include both males and females. Mean +/− S.E.M. is depicted. *p *<* 0.05, repeated measures ANOVA, Bonferroni multiple comparisons test. ^#^*p* *<* 0.05, paired *t*-test, min −1 versus min 0.

Distance traveled in response to the white noise control stimulus did not differ between groups and both genotypes exhibited behavioral inhibition (i.e., a reduction in motion following the noise control) (Fig. 3a genotype *F*_(1, 68)_ = 2.548, *p* > 0.05). As shown by our laboratory and others previously^15,18–22^, a striking increase in social exploratory behavior (i.e., distance traveled) was observed in response to playback of pro-social 50-kHz USV. Distance traveled increased in response to playback of 50-kHz USV (i.e., higher during playback compared to the minute prior) in *Ube3a*^*m+/p+*^ and *Ube3a*^*m−/p+*^ rats (Fig. 3b paired *t*-test *Ube3a*^*m−/p+*^: *t*_(1, 38)_ = 5.271, *p* < 0.0001 and paired *t*-test *Ube3a*^*m+/p+*^: *t*_(1, 14)_ = 4.94, *p* < 0.0001, respectively), as expected. Interestingly, however, the magnitude of the distance increase in the *Ube3a*^*m−/p+*^ juvenile rats was significantly lower than in the wildtype *Ube3a*^*m+/p+*^ littermates (Fig. 3b genotype *F*_(1, 68)_ = 4.908, *p* < 0.04, Bonferroni correction *p* < 0.05, for minutes 1 and 2 post call play).

Social exploratory behavior displayed by *Ube3a*^*m+/p+*^ rats was clearly directed towards playback of pro-social 50-kHz USV, as reflected in the more sensitive parameter of time spent on the arms proximal to the sound source emitting 50-kHz USV. *Ube3a*^*m+/p+*^ rats spent significantly longer on the arms proximal to the speaker emitting the 50-kHz USV upon playback and for minutes afterwards (Fig. 3c*F*_(1, 60)_ = 7.471, *p* < 0.01, Bonferroni-corrected *p* < 0.05 for minutes 1, 2, and 5). In contrast, the *Ube3a*^*m−/p+*^ rats failed to show a sustained preference and only spent significantly more time on the proximal arms during the minute of the USV playback (Fig. 3d*F*_(1, 60)_ = 3.380, *p* > 0.05). Fig. 3e, f show representative heat maps of the distance and direction traveled in response to the 50-kHz stimuli by *Ube3a*^*m+/p+*^ and *Ube3a*^*m−/p+*^ respectively. These figures illustrate the striking increase in directed social exploratory behavior quantified in Fig. 3c and the lower, atypical pattern of social exploration quantified in Fig. 3d.

### *Ube3a*^*m−/p+*^ but not *Ube3a*^*m+/p*−^ illustrate neuroanatomical pathology at PND 21

**Fig. 4:**
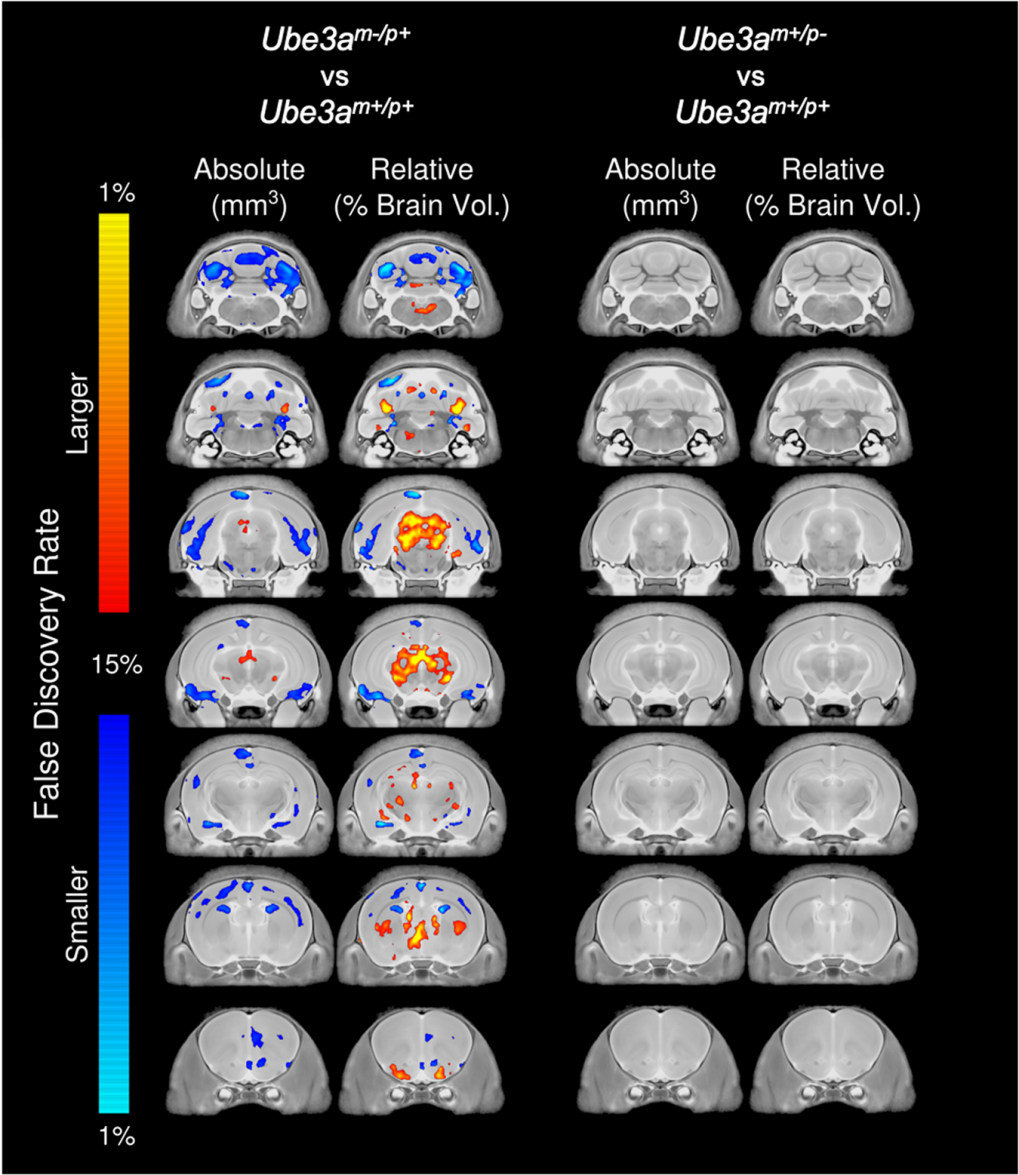
Neuroanatomical pathology in *Ube3a*^*m−/p+*^ rats at PND 21. Representative coronal slice series highlighting regional brain differences in absolute (mm^3^) and relative (%) brain volume between *Ube3a*^*m*−/*p*+^ and *Ube3a*^*m+/p+*^ (left) and between *Ube3a*^*m+/p−*^ and *Ube3a*^*m+/p+*^ (right). Regions with decreased volume in *Ube3a*^*m*−/p+^ include the cerebral cortex, cerebellum, and amygdala. Regions with increased volume in *Ube3a*^*m−/p+*^ include the periacqueductal gray, thalamus, and hypothalamus. *Ube3a*^*m+/p−*^ did not exhibit altered neuroanatomy compared to wildtype. Analyses include both males and females.

Total brain volumes were not observed to be different between groups, but there was a trend found in the difference between the *Ube3a*^*m−/p+*^ and *Ube3a*^*m+/p+*^ littermates (−3.5% in *Ube3a*^*m−/p+*^, *p* = 0.06, *q* = 0.10). Structural differences were further examined on a regional and voxelwise level. In the *Ube3a*^*m−/p+*^ rats, several regional differences were observed voxelwise in both absolute and relative volume at a false discovery rate (FDR) of *q* < 0.15 (Fig. 4). Regionally, when the 98 different regions were examined, there were no differences found in absolute volume for the full group, males, or females. However, there were several trends in the combined group. The majority of the 98 different regions were decreased in size in comparison to their wildtype counterparts by −3 to −5%. This led to trends in several different areas of interest. For example, the primary motor cortex appears to be smaller in the *Ube3a*^*m−/p+*^ rats (−3.6%, *p* = 0.046, *q* = 0.10), however, this effect is not observed upon controlling for multiple comparisons. Similar findings were seen in the cerebellum, with the cerebellar lobules decreased in size by −3.8 to −4.6% and *p*-values ranging from 0.03 to 0.05; however, again these trends were not considered significant based on FDR thresholds (Supplementary Table S3). Dividing the groups into different sexes revealed that the females seemed to be driving these volume differences; however, no sex by genotype interaction was found to be statistically significant. In contrast to these results, when examined on a regional or voxelwise basis, no differences were present between *Ube3a*^*m+/p−*^ rats and their *Ube3a*^*m+/p+*^ littermates, highlighting a distinct phenotype based on parental allele inheritance, as expected given the genetic paternal imprinting of *Ube3a*.

### *Ube3a*^*m−/p+*^ rats exhibit deficits in touchscreen discrimination learning and memory

**Fig. 5:**
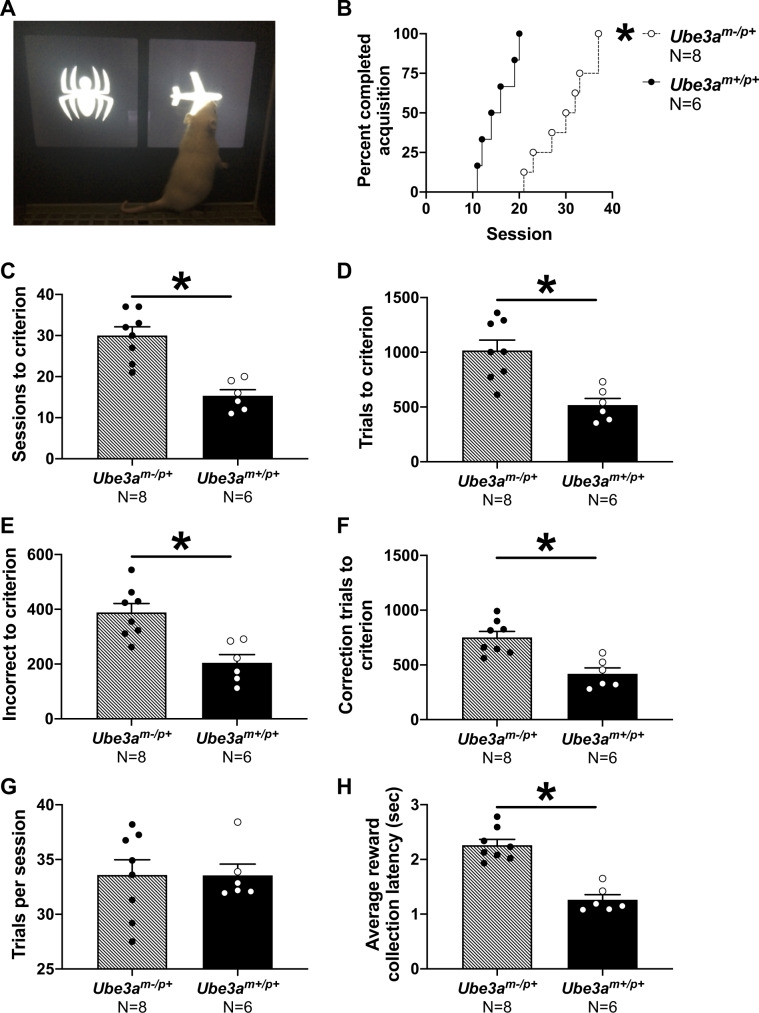
Delayed touchscreen learning in *Ube3a*^*m−/p+*^ rats. **a** Representative image of a subject rat performing the touchscreen pairwise discrimination. **b** *Ube3a*^*m−/p+*^ adult rats took significantly longer to learn the correct response compared to wildtype littermates, requiring **c** more sessions, **d** more trials, **e** more incorrect responses, and **f** more correction trials to reach criterion. **g** The average number of trials completed per session did not differ between genotypes. **h** *Ube3a*^*m−/p+*^ adult rats took longer to collect the pellet after responding correctly. Mean + S.E.M. is depicted. **b**: *p *<* 0.0001, Log-rank (Mantel-Cox test). **c**–**h**: *p *<* 0.05, Student’s *t*-test.

Given a limited number of testing chambers, for feasibility our initial touchscreen experiments were selectively performed in males. *Ube3a*^*m−/p+*^ required significantly more training days to learn to discriminate two images displayed on the touchscreen. Representative task images are shown in Fig. 5a. Analysis of survival curves (i.e., percentage of rats that reached the 80% accuracy criterion on each training day) indicated that *Ube3a*^*m+/p+*^ wildtype control male rats took about 10–15 sessions to reach criterion while *Ube3a*^*m−/p+*^ rats took about 20–40 sessions (Fig. 5b Log-rank (Mantel-Cox) test: Chi square = 15.70, df = 1, *p* < 0.001). Analysis of additional parameters indicated that *Ube3a*^*m−/p+*^ rats illustrate robust learning and memory impairments. *Ube3a*^*m−/p+*^ rats took more sessions (Fig. 5c*t*_(1, 12)_ = 5.281, *p* < 0.001), required more trials (Fig. 5d*t*_(1, 12)_ = 4.055, *p* < 0.002), required a greater number of incorrect responses (Fig. 5e*t*_(1, 12)_ = 4.003, *p* < 0.002), and needed more correction trials (Fig. 5f *t*_(1, 12)_ = 4.255, *p* < 0.002) to reach the learning criterion. Motivational control parameters such as numbers of trials completed per session did not differ between genotypes, also showing no motor impairments (Fig. 5g*t*_(1, 12)_ = 0.018, *p* > 0.05). Panel h is the latency or time it takes for the rat to collect its pellet after responding correctly. *Ube3a*^*m−/p+*^ rats took approximately twice as long as wildtypes to collect rewards (Fig. 5h*t*_(1, 12)_ = 6.918, *p* < 0.0001). Learning and memory as assessed in the novel object recognition test was not affected (Fig. S4). Both *Ube3a*^*m−/p+*^ and *Ube3a*^*m+/p+*^ rats spent significantly more time investigating the novel object than the familiar object as determined by automated tracking (Fig. S3a*Ube3a*^*m−/p+*^ *t* = 4.428, df = 80, *p* < 0.001; *Ube3a*^*m+/p+*^ *t* = 5.162, df = 60, *p* < 0.001) and hand-scoring (Fig. S3b*Ube3a*^*m−/p+*^ *t*_(1, 80)_ = 5.005, *p* < 0.001; *Ube3a*^*m+/p+*^ *t*_(1,60)_ *=* 4.832, *p* < 0.001).

## Discussion

Genetically engineered rat models are becoming a widely feasible investigative approach for preclinical research that retains a high degree of genetic conservation relative to humans for targeted therapeutic development while providing enhanced behavioral capabilities relative to mice. In addition to the species advantages, this is the first model to be null for the entire *Ube3a* gene. Since the gene that is causal in AS is known to be *UBE3A*, several innovative gene correction strategies are being pursued for AS and *UBE3A*/UBE3A replacement. In order to measure whether therapeutic methods have efficacy, clear and robust functional phenotypes are required. The results presented here address this unmet need in AS research by providing a novel rat model system with a complete gene deletion and quantifying behavioral and anatomical characteristics that can be used to test the efficacy of therapeutics.

Gene targeted therapeutic approaches, including antisense oligonucleotides^23^, viral vector delivery^24^, and artificial transcription factors (ATFs)^25,26^, are being tested for application to AS. Other innovative methodologies that are currently being pursued include gene-modifying CRISPR-dCas9 and cross correction of UBE3A by hematopoietic stem cells^27^. In addition to UBE3A targeted therapies, numerous dietary and traditional pharmaceutical treatments have shown alleviation of one or more symptom domains found in the mouse model, including but not limited to ketone esters^28^, dietary methylation, ErbB inhibitors^29^, and topoisomerase inhibitor drugs^30^. Behavioral rescues have been reported utilizing the original exon-2 deletion mouse from Jiang and Beaudet backcrossed onto the C57BL6/J^23,31,32^ and maintained by the Jackson Laboratory, or a genetic-based tamoxifen-induced conditional hypomorphic line on a 129-based mixed background backcrossed to a sub-strain of C57BL6 in the Elgersma group^33–35^. Reports of rescue have relied heavily on outcomes of the rotarod, marble-burying, elevated plus and zero maze tasks of conflict anxiety, and contextual fear conditioning. These tasks are well standardized, but may lack translational predictive value and may underlie some of the large gap between basic scientific research and translation to therapeutics, given the lack of compound approval for the AS community to date.

Our results illustrate a robust phenotype and impairment in developmental ultrasonic vocalizations (USV), a clear social communication readout in models of neurodevelopmental disorders (NDD). Some Angelman syndrome mouse models have demonstrated USV dysregulation. One study reported increased USV emission. The reported results were highly dependent on the mouse’s inbred background strain, and conclusions varied across publications^36,37^. Our results, which highlight reduced USV emission, were reproduced by two independent laboratories (UC Davis MIND and Baylor College of Medicine) and align with the AS clinical profile of reduced communication. Moreover, given the outbred nature of the rat over the mouse, phenotypes are likely more generalizable by inherent natural genetic variation, which is proving to be a successful approach in neurodegenerative disorder research^38^. Signal to noise ratio and assay sensitivity have plagued bench-to-bedside drug development efforts across neurodevelopmental and neurodegenerative disorders, an issue that may be alleviated by this novel rodent species approach.

We discovered impairments in juvenile behavioral responses to the playback of 50-kHz USV, a positive affiliative social contact call associated with play and social interactions, in the *Ube3a*^*m−/p+*^ rats. Social exploratory and approach behaviors, typically evoked by the social contact calls, were weak in rats with the *Ube3a*^*m−/p+*^ deletion. They did not exhibit as highly elevated distance traveled nor extended durations of the behavioral response compared to that of wildtype littermate controls. Wildtype rats spent significantly longer on the arms proximal to the speaker upon initial playback of the 50-kHz USV (i.e., hearing it) and for minutes after the 50-kHz USV stopped being played (i.e., after hearing it), suggesting that they kept searching for the conspecific even after 50-kHz USV emission ended. In contrast to the wildtype, *Ube3a*^*m−/p+*^ subjects failed to show a strong, sustained response to hearing the cue and only spent significantly more time on the proximal arms during the time period the audio cue was “on”. This was an unusual display of social behavior as there should be (1) a clear behavioral preference for the proximal versus distal arms and (2) sustained attempts to locate the source of the emission, as previously reported across genetic models and groups^12,15,18,20,39^. The behavioral alterations displayed by *Ube3a*^*m−/p+*^ rats in response to pro-social 50-kHz USV were clear and substantially more prominent than the social communication deficits seen in other recently developed rat models relevant to autism, including *Shank3*^15^ and *Cacna1c*^18,19^. While individuals with AS are typically thought of as being highly social, and/or uninhibited socially, with excessive smiling being a hallmark behavioral feature in AS, aberrant social behavior is considered a core phenotypic trait. Thus, the absence of typical social approach to the acoustic call is an interesting discovered abnormality. Of additional potential will be the in-depth investigation of whether the social communication deficit shall be attributed to a lack of understanding of the 50-kHz USV call (i.e., resulting from cognitive circuit disruption) or a requirement for longer bouts of stimulation than tested on this initial series of experiments (i.e., resulting from impairments in motivation circuits). Other testable hypotheses include impaired sustained attention. Future directions will be focused on the detailed nuance of this social behavior and deciphering the response calls during the playback assay. These functional findings of reduced USV and response to USV are critical to the AS community in positioning for an FDA approved clinical trial with a communication outcome, now that two preclinical developmental time points, postnatal life and juveniles, illustrate preclinical deficient USV. This lower signal in communication will open new opportunities to test therapeutics as most individuals with AS do not ever develop speech or more than a few vocalizations^40–42^.

Movement disorders affect nearly every individual with AS^40,41,43,44^. The most common motor problems include spasticity, ataxia of gait (observed in the majority of ambulatory individuals), tremor, and muscle weakness^45^. We observed motor deficits in vertical rearing at two different time points across development, however gross overall motor ability to navigate an open field was unaltered. Low counts in vertical rearing may indicate hindlimb weaknesses. We also observed motor coordination dysfunction in young subjects on the rotarod. Motor deficits have been key in the study of mouse models of AS, as dysfunction on the rotarod and fewer rearing movements have been one of the most consistently reported motor behavioral phenotypes when using the C57BL6/J^46–48^ but not in the 129/SvEv background strain AS mice. Observation of these motor deficits in the genetically heterogenous rat model extends the theory that preclinical data can generalize to genetically diverse clinical populations^49^. A secondary advantage of this unique motor phenotype in rat models is the added value of a larger species. While *Ube3a*^*m−/p+*^ rats show motor phenotypes that support its use for future pharmacological/gene therapy rescue studies, these hindlimb coordination motor deficits do not confound other behavioral measures such as social communication and learning and memory. Motor confounds have plagued the interpretation of complex behaviors for numerous neurodevelopmental disorder mouse models, such as those of Phelan-McDermid and Timothy syndromes^50–52^, yet by using a larger species, motor deficits can be delineated and detected without affecting other measures.

Our imaging data highlighted a wide variety of volumetric abnormalities. The majority of regions decreased in size from 3 to 5%. Several differences were seen at *q* < 0.10, including, but not limited to, the cortex, several white matter structures, and the cerebellum. This does highlight the expected microcephaly although merely by a trend. Our full group showed 3.5% reduction but only was *p* = 0.06, *q* = 0.10, leading to our conclusion of no differences in total brain volume at PND 21. This could be the result of the juvenile age at image acquisition as clinically microcephaly develops over the first 3 years of life in AS individuals and is not 100% penetrant^43,53^. Fiber tracts throughout the brain show an insignificant loss in size in the *Ube3a*^*m−/p+*^ rats (−3.2% in the corpus callosum, −4.0% in the fimbria, and −4.3% in the arbor vita of the cerebellum). However, when the total brain volume is accounted for, there is a slight increase in the volume of the fiber tracts, particularly in the fimbria, fornix, and cerebral peduncle. Interestingly, the effect on the white matter at postnatal day 21 appeared minimal in the *Ube3a*^*m−/p+*^ rats, in contrast to what has been shown in adult exon-2 deletion mice^54^. While the overall cortical losses seem to be consistent between our work and the earlier mouse study, Judson et al. report volume losses of 11–13% in several white matter regions throughout the brain of *Ube3a*^*m−/p+*^ mice, whereas we discovered modest 2–5% differences here for the same white matter tracts. While the white matter findings were not as drastic as those shown in the previous work with mice, it will be interesting to examine the rats using diffusion tensor imaging (DTI) to see if that technique is more sensitive to the white matter differences seen with the mouse model. In comparison to other rodent mouse models related to intellectual disability and autism, the *Ube3a*^*m−/p+*^ seems to closely resemble the Magel2 mouse^55,56^, which also displayed a 3.4% decrease in total brain volume and similar volume differences in structures throughout the brain ranging from −4 to 5%, including the parietotemporal lobe, the amygdala, and the dentate gyrus of the hippocampus. All of these regions had similar differences on the order of −3 to 5% in the *Ube3a*^*m−/p+*^ rat but did not reach significance at current thresholds. This is likely due to the increased variability for regions in the outbred rat versus a congenic wildtype C57Bl/6J mouse. In an average region in a wildtype C57Bl/6J mouse one standard deviation for a combined sex group of 20 mice is ~6%, but in the wildtype rat here it is ~8.4%. A recent clustering analysis by Ellegood et al. examined 26 different mouse models related to autism and clustered them into three different groups^57^. Group 2 in that study was characterized by smaller cortical and white matter structures throughout the brain consistent with what has been shown here in the *Ube3a*^*m−/p+*^ rat model. Other models in Group 2 were the 15q11-13 duplication^58^, Itgb3^59^, Slc6a4 Ala56 KI^60^, and the humanized Androgen Receptor mouse^61^.

Learning and memory impairments have been observed in some but not all studies of *Ube3a* mutant mouse models depending on the background strain and age at time of testing^46,47^. The preponderance of these findings used standard assessments such as the electrophysiological correlate of learning and memory, long-term potentiation, and behavioral assays, including Morris water maze and contextual fear conditioning^29,62–67^. Cognitive dysfunction was also postulated by enhanced operant extinction^68^ and touchscreen visual discrimination in the exon-2 deletion mouse model^67^, albeit this exon-2 line has prominent motor deficits that complicate delineation of cognitive versus motor. Moreover, frequently, learning and memory deficits in the mouse model were not observed or reproduced. We expanded and improved the translational value by using computerized-based touchscreen technology and illustrating robust deficits in visual discrimination of two novel equi-luminescent stimuli in *Ube3a*^*m−/p+*^ rats. The discrimination deficit in the *Ube3a*^*m−/p+*^ rats has translational value via the touchscreen methodology, which is utilized by clinicians using Cogmed™ or the NIH Toolbox® computerized-based testing batteries for many domains of learning and memory and executive function in several genetic NDD^69–71^, and will allow for testing of cognitive enhancing agents, as well as the cognitive domain by gene therapies. Our data make an interesting observation in the *Ube3a*^*m−/p+*^ rat beyond the obvious learning deficit: we also saw longer timings to collect food rewards upon correct responses, which has been suggested as evidence of impaired motivational circuity. While one metric of impaired motivation may be a fluke, this elevated latency to collect reward was supported by the lack of social approach in the playback assay. Combined, these data make a stronger statement about reduced motivation in the AS rat model. Of course, this is our first characterization and we will have to perform more assays specific to the motivational domain in order to more definitively make this conclusion. We have also been trying differing flavors of pellet rewards (chocolate, banana, sucrose) to gather more data on motivational components of the behavioral deficits, as motivation is clearly not a problem in the AS clinical population for neither learning nor social assessments.

The pipeline of translation from preclinical studies to clinical trial is highly unique and varies greatly depending on the type of therapy (pharmaceutical, biological, genetic therapy) and prior research performed. For example, some traditional medicines may be re-purposed whensafety data is already published and known, however, for precision medicine genetic therapies, more work on the safety and tolerability end is required. In our experience, to date, the first steps are to show functional efficacy of the compound in a preclinical model and to illustrate a lack of toxicity and sufficient safety in a secondary species. Then, our ability to manufacture said novel therapeutic at levels of human doses, generated in a good manufacturing process facility, needs to be demonstrated. This, combined with therapeutic profile, pharmacokinetics, pharmacodynamics, and therapeutic kinetics would be put together for an innovative drug discovery (IND) application for the Food and Drug Administration (FDA) for clinical trial approval. Currently for AS, preclinical testing is ongoing for viral vectors, antisense oligonucleotides, artificial transcription factors, and stem cell delivered viral vectors and proteins, as well as simpler therapeutics from pharmaceutical companies. Each has a unique pathway to clinical trial.

Going forward, for successful translation to clinical trials, targeted treatments need to improve functional behavioral outcomes relevant to Angelman syndrome to improve the likelihood of translational success and receive FDA approval to conduct a clinical trial. Our report describes, for the first time, a novel model for Angelman syndrome that exhibits translationally relevant functional behavioral and anatomical outcomes resulting from a full deletion of *Ube3a*. The data presented are therefore highly relevant and important for the advancement of testing genetic and pharmacological therapeutics for Angelman syndrome.

## Methods

### Subjects

All animals were housed in a temperature-controlled vivarium maintained on a 12:12 light–dark cycle. All procedures were conducted in compliance with the NIH Guidelines for the Care and Use of Laboratory Animals and approved by the Institutional Animal Care and Use Committee of UC Davis. *Ube3a*^*m+/p−*^ males were bred with wildtype (*Ube3a*^m+/p+^) Sprague-Dawley females purchased from Envigo (East Millstone, New Jersey, USA) in a conventional rat vivarium at UC Davis. The resulting female paternally inherited rats (*Ube3a*^*m+/p−*^) and male wildtype (*Ube3a*^*m+/p+*^) rats were paired for breeding to generate maternally inherited mutants (*Ube3a*^*m−/p+*^) and wildtype (*Ube3a*^*m+/p+*^) offspring for behavioral and anatomical testing. Male paternally inherited mutant (*Ube3a*^*m+/p−*^) and female wildtype (*Ube3a*^*m+/p+*^) rats were also paired for breeding to generate paternally inherited rats (*Ube3a*^*m+/p−*^) and wildtype (*Ube3a*^*m+/p+*^) for colony maintenance and control testing. To identify rats, pups were labeled via paw tattoo on postnatal day (PND) 2 with non-toxic animal tattoo ink (Ketchum Manufacturing Inc., Brockville, ON, Canada). A 23-gauge needle was used to subcutaneously insert the ink into the center of the paw. Rats were additionally identified at weaning via tail-marks made with permanent marker. Tattoos and tail-marks were coded to allow investigators to run and score behaviors blind to genotype. At PND 2, tissue samples were collected for genotyping via a small tail snip. Genotyping was performed with REDExtract-N-Amp (Sigma Aldrich, St. Louis, MO, USA) using primers Rube1123 TAGTGCTGAGGCACTGGTTCAGAGC, Rube1606r TGCAAGGGGTAGCTTACTCATAGC, Ub3aDelSpcfcF6 ACCTAGCCCAAAGCCATCTC, and Ub3aDelR2 GGGAACAGCAAAAGACATGG.

### Western blots

Rats were cervically dislocated and discrete brain structures were rapidly removed using a 4 × 4 mm matrix. Protein was extracted using RIPA buffer + 1% protease inhibitor. Extracted protein was quantitated using BCA assay (ThermoFisher, Waltham, MA). Forty micrograms of protein was denatured with 5x loading dye (National Diagnostics) for 5 min at 95 °C and separated on a 10% Bis-Tris Gel (BioRad, Hercules, CA). Overnight transfer was performed at 30 V to polyvinylidene difluoride (PVDF) membranes (Invitrogen, Carlsbad, CA). PVDF membranes were blocked for 45 min with Tris-buffered saline with Tween (TBST) (1x TBS + 0.1% Tween-20) with 5% seablock. Following blocking, PVDF membranes were incubated with ms-UBE3a (1:1000, Sigma 8655) and rb-beta Tubulin (1:2000) in 5% seablock TBST for 2 h at room temperature (RT). Following incubation, membranes were washed with TBST 3x for 5 min. PVDF were then incubated with Donkey anti-mouse LICOR 680 (1:2000) and donkey anti-rabbit LICOR 800 (1:2000) in 5% seablock TBST for 2 h at RT. Following incubation, gels were washed 2x with TBST before being stored in 1x TBS. PVDF membranes were imaged on a LICOR Odyssey. Densitometry analysis were performed using ImageJ (NIH, Bethesda, MD).

### Cohort 1 behavioral assays

#### Pup ultrasonic vocalizations (USV)

On PND 4, 6, 8, 10, 12, 14, 16, and 18, isolation-induced USV were collected for 3 min as previously described^15^. Each pup, randomly selected from the nest, was placed in a small container with clean bedding and calls were recorded within a sound-attenuating chamber using an ultrasonic microphone and Avisoft-RECORDER software (Avisoft Bioacoustics, Glienicke, Germany). Immediately following, body temperature and weight were measured. Call spectrograms were displayed using Avisoft-SASLab Pro and counted manually by a trained investigator blind to genotype. Pup calls were also collected at Baylor College of Medicine on PND 8 for 2 min over the 3 min protocol used at the UC Davis facility. Calls were recorded within a sound-attenuating chamber using an ultrasonic microphone and Noldus Ultravox XT 3.2 (Noldus, Wageningen, The Netherlands).

#### Developmental milestones

In a separate group of animals, on PND 4, 6, 8, 10, 12, 14, 16, and 18, developmental milestones were assessed as described previously^1^. Body weight, body length, tail length, and head width were measured with a scale and sliding ruler. Righting reflex was tested by placing each pup on its back and measuring the time taken to flip over onto all four paws. The average of two trials was recorded. Circle traverse was tested by placing each pup in the center of a circle (12.5 cm *d*) and measuring the time taken to fully exit the circle. Cliff avoidance was tested by placing each pup near the edge of a table, with its nose just beyond the edge, and measuring the time taken to make a 90 degree turn away from the cliff, thereby becoming parallel with the table edge. Each pup was allotted 30 sec to complete each task and failure to complete a task was recorded as the maximum score of 30 sec. The first day in which rooting reflex, forelimb grasping, and bar hold were demonstrated was recorded. Rooting reflex was measured as a turn of the head to whisker stimulation. Forelimb grasping was measured as grasping of a bar being moved upward along both front paws. Bar hold was measured as a pup’s ability to hold onto a bar with their front paws and support their body weight for at least ten seconds. Day of eye opening was also recorded.

#### Open field locomotion

At PND 19 and again at PND 39–44, exploratory activity in a novel open arena was evaluated as described previously^15,72^. Each animal was placed in an Accuscan Animal Activity Monitor (Omnitech Electronics, Columbus, OH, USA), which automatically measured beam breaks for cm^2^ of movement via horizontal activity, vertical activity, time in center, and total distance moved over a 30-min session.

#### Novel object recognition

At PND 45–53, novel object recognition was assessed using methods similar to those described previously^51,73,74^. Rats were given 30 min to freely explore an empty arena (54.1 cm *l* × 54.1 cm *w* × 34.3 cm *h*) on 2 consecutive days. After the second exploration session, two identical objects were placed in the arena with the subject and the rat was allowed 10 min to investigate and become familiar with the objects. Following a 60-min isolation, the rat was placed back in the arena (clean) with one familiar and one novel object (both clean) and allowed 5 min to investigate.

#### Touchscreen pairwise discrimination

Starting at PND 65–72, pairwise visual discrimination was tested in an automated Bussey-Saksida touchscreen system (Lafayette Instrument, Lafayette, IN, USA) using a procedure modified for rats from those described previously in mice^50,75–77^. Rats were food restricted to 85% of their free-feeding weight. An efficient pre-training procedure based on previously published work was utilized. The pre-training consisted of five stages to train rats to touch the screen, collect the reward, and initiate trials. Stage 1 consisted of a 20-min habituation to the chamber and the sucrose pellet reinforcer with no light or images on the screen. All following sessions lasted 30 min. During Stage 2, three sucrose pellets were dispensed upon the screen being touched, and one pellet was dispensed if the screen was not touched. Stage 2 lasted 5 days, until rats completed an average of 30 trials in the 30 min session. During Stage 3, one sucrose pellet was dispensed upon the screen being touched, and no pellets were dispensed if the screen was not touched. Stage 3 lasted 2 days, until rats completed an average of 30 trials during the 30 min session. During Stage 4, rats were required to initiate each trial by entering and exiting the food magazine. One sucrose pellet was dispensed upon the screen being touched, and no pellets were dispensed if the screen was not touched. Stage 4 lasted 1 day, until rats completed an average of 30 trials in the 30 min session. During Stage 5, a random image from a set of 40 images was presented in one of the windows until the screen was touched. One pellet was dispensed if the image was touched, while touching the blank side was discouraged by no reward and by a 5-sec timeout during which an overhead light was turned on. Stage 5 lasted 3 days, until every rat completed at least 30 trials with an average accuracy of at least 80% over two consecutive sessions. Images used in Stages 4 and 5 were not used in the subsequent pairwise visual discrimination task and successful completion of all five stages of pre-training was required for participation in the discrimination task. Rats were trained to discriminate between two novel images (spider and plane) displayed in two side-by-side windows in a pseudo-randomized order. Each 30 min session consisted of an unlimited number of trials separated by a 20-sec intertrial interval. The image designated as correct was counterbalanced across rats within each genotype. Touching the correct image was rewarded with a sucrose pellet while touching the incorrect image was discouraged with no pellet and a 5-sec timeout with the light on. Incorrect responses were immediately followed by correction trials in which the images were presented in the identical manner to the previous trial until the rat selected the correct image. Successful acquisition was defined as achieving at least 80% correct responses over two consecutive sessions with a minimum of 30 trials completed during each 30 min session.

### Cohort 2 behavioral assays

#### Developmental milestones

On PND 4, 6, 8, 10, 12, 14, 16, and 18, developmental milestones were assessed as described previously^73^. Negative geotaxis was tested by placing each pup on an angled screen (45 degrees) facing downwards and measuring the time taken to make a complete 180 degree turn up the screen. The maximum time allowed was 30 sec.

#### Playback of pro-social 50-kHz USV

On PND 26–33, behavioral response to playback of pro-social 50-kHz USV was used to identify if *Ube3a*^*m−/p+*^ would exhibit similar social exploratory behaviors as *Ube3a*^*m+/p+*^ in response to social contact calls. The procedure was performed as previously described^15^. All rats were handled for 2 days prior to testing in a standardized manner (5 min per rat per day). Social exploratory and approach behavior in response to playback of pro-social 50-kHz USV was assessed on an elevated radial eight-arm maze (48.0 cm above floor; arms: 40.0 cm *l* x 10.0 cm *w*) surrounded by a black curtain under indirect dim white light (8 lux) according to a modified protocol previously established^12,15,19^. Acoustic stimuli were presented through Ultra-SoundGate 116 Player (Avisoft Bioacoustics) connected to an ultrasonic loudspeaker (ScanSpeak, Avisoft Bioacoustics) placed 20 cm away from the end of one arm. An additional, but inactive loudspeaker was arranged symmetrically at the opposite arm as a visual control. Two acoustic stimuli were used: (1) pro-social 50-kHz USV and (2) White Noise; the latter serving as a time- and amplitude-matched acoustic stimulus control^20^. Pro-social 50-kHz USV used for playback were recorded from a naive male rat during exploration of a cage containing scents from a recently separated cage mate. The 50-kHz USV stimulus consisted of 221 natural 50-kHz USV (total calling time: 15.3 sec), composed of a sequence of 3.5 sec, which was repeated for 1 min, that is, 17 times, to assure the presentation of a high number of frequency-modulated calls within a relatively short period of time. After an initial 15-min habituation period, each rat was exposed to 1-min playback presentations of 50-kHz USV and White Noise, separated by a 10-min inter-stimulus interval. Stimulus order was counterbalanced to account for possible sequence effects. The session ended after an additional 10-min post-stimulus phase (total test duration: 37-min period). Behavior was monitored by a video camera mounted 1.7 m centrally above the arena and analyzed using EthoVision XT 10 (Noldus, Wageningen, The Netherlands). Distance traveled served as a measure for locomotor activity. Time spent on arms proximal and distal to the active ultrasonic loudspeaker served as measures for stimulus-directed locomotor activity^20^.

#### Accelerating rotarod

To corroborate and reproduce the Nash laboratory report, at PND 43–45, motor coordination, balance, and motor learning were tested with an accelerating rotarod (Ugo Basile, Gemonio, Italy) as described previously^2,3^. Rats were placed on a rotating cylinder that slowly accelerated from 5 to 40 revolutions per min over 5 min. Rats were given three trials per day with a 45–60-min intertrial rest interval and tested for 3 consecutive days for a total of nine trials. Performance was scored as latency to fall off the cylinder with a maximum latency of 5 min.

#### Adhesive removal

At PND 52, a task of adhesive removal was used to assess sensory and find motor ability using a previously described mouse protocol modified for rats^78^. Rats individually habituated to an observation arena for 10 min and then a small round adhesive sticker (0.64 cm *d*; Avery Products Corporation, Strongsville, OH) was placed on the forehead. The latency to initiate removal of the sticker and the total elapsed time until complete removal of the sticker were recorded.

### Magnetic resonance imaging

A multi-channel 7.0 Tesla MRI scanner (Agilent Inc., Palo Alto, CA) was used to image the rat brains within their skulls. Seven brains were scanned in one session, using an array of millipede coils and a T2 weighted 3D Fast Spin Echo Sequence (FSE) with an echo train length of 12 and a cylindrical sampling of k-space to reduce acquisition time^79^. Other sequence parameters included: TR of 350 ms, echo spacing of 10.5 ms, with the center of k-space acquired in successive averages on the 5^th^ and the 6^th^ echo, FOV of 3.6 × 3.6 × 4.0 and a matrix size of 456 × 456 × 504, yielding an image resolution of 79 μm isotropic. Total imaging time for this protocol is ~3 h and 20 min.

To visualize and compare any changes in the rat brains, the images are linearly (6 followed by 12 parameters) and non-linearly registered together. Registrations were performed with a combination of mni_autoreg tools and ANTS (advanced normalization tools)^80,81^. All scans are then resampled with the appropriate transform and averaged to create a population atlas representing the average anatomy of the study sample. The result of the registration was to deform all images into alignment with each other in an unbiased fashion. For the volume measurements, this allowed us to analyze the deformations needed to take each individual rat brain’s^82^ anatomy into this final atlas space, the goal being to model how the deformation fields relate to genotype. The Jacobian determinants of the deformation fields were then calculated as measures of volume at each voxel. Significant volume changes could then be calculated by warping a pre-existing rat MRI atlas onto the population atlas. An open-source classified atlas for the Fischer-344 rat brain has been created and maintained by the Near Lab at the Douglas Institute in Montreal, QC (https://www.nearlab.xyz/fischer344atlas). This segmented atlas allowed us to assess the volume of 98 different segmented structures^83^, encompassing the cortex, large white matter structures (i.e., corpus callosum), ventricles, cerebellum, brain stem and olfactory bulbs in all brains. Further, these measurements could be examined on a voxelwise basis to localize the differences found within regions or across the brain. Multiple comparisons in this study were controlled for using the false discovery rate^84^. We reported combined sex results in the main text.

### Statistical analysis

Developmental assays were analyzed with two-way repeated measures ANOVA, with genotype as the between-group factor and time as the within-group factor. Touchscreen parameters (sessions to reach criterion, trials to criterion, errors to criterion, and correction trials to criterion) were analyzed with unpaired (Student’s) *t*-test. Log-rank (Mantel-Cox) test was used to analyze the percentage of animals that reached criteria in the survival/completion analysis for the touchscreen test. Open field parameters (horizontal activity, vertical activity, and center time) were analyzed with two-way repeated measures ANOVA, with genotype as the between-group factor and time as the within-group factor. Comparisons between time sniffing the novel object were compared within each genotype, as previously described^73,85^. Data were analyzed with Graphpad Prism. All significance levels were set at *p* < 0.05 and all *t*-tests were two-tailed. Groups sizes were chosen based on past experience and power analyses^86^. Significant ANOVAs were followed by Bonferroni-Dunn or Holm-Sidak posthoc testing. Behavioral data passed distribution normality tests, were collected using continuous variables, and thus were analyzed via parametric analysis in all assays. For all behavioral analyses, variances were similar between groups and data points within 2 standard deviations of the mean were included in analysis. For the MRI analysis, separate linear models were measured for both absolute and relative regional and voxelwise volumes. Additionally, a final linear model was used to determine if there were any sex by genotype interactions. In all cases, multiple comparisons were controlled for using the false discovery rate^84^. Anatomical results reported combined both sexes.

## Supplementary information

Supplementary Figures 1-4 and Tables S1 and S2

Supplementary Table S3
