# Supplementary material for: Translational outcomes in a full gene deletion of ubiquitin protein ligase E3A rat model of Angelman syndrome
Source: Transl Psychiatry. 2020 Jan 27;10:39. doi: 10.1038/s41398-020-0720-2 (PMC7026078; doi:10.1038/s41398-020-0720-2)
Supplement: Supplementary file 1 — Supplementary Figures 1-4 and Tables S1 and S2 [file 41398_2020_720_MOESM1_ESM.docx]

**Supplementary Figure S1. Schematic of the Angelman syndrome rat model.** The rat wildtype (WT) locus encompassing the Ube3a coding region is shown on top. Using the CRISPR/Cas9 system the ~90 kb *Ube3a* gene region was deleted and the genomic junction of the *Ube3a* knock-out allele was determined by Sanger sequencing. Genomic sequence of the *Ube3a* knockout allele is shown in the lower panel.


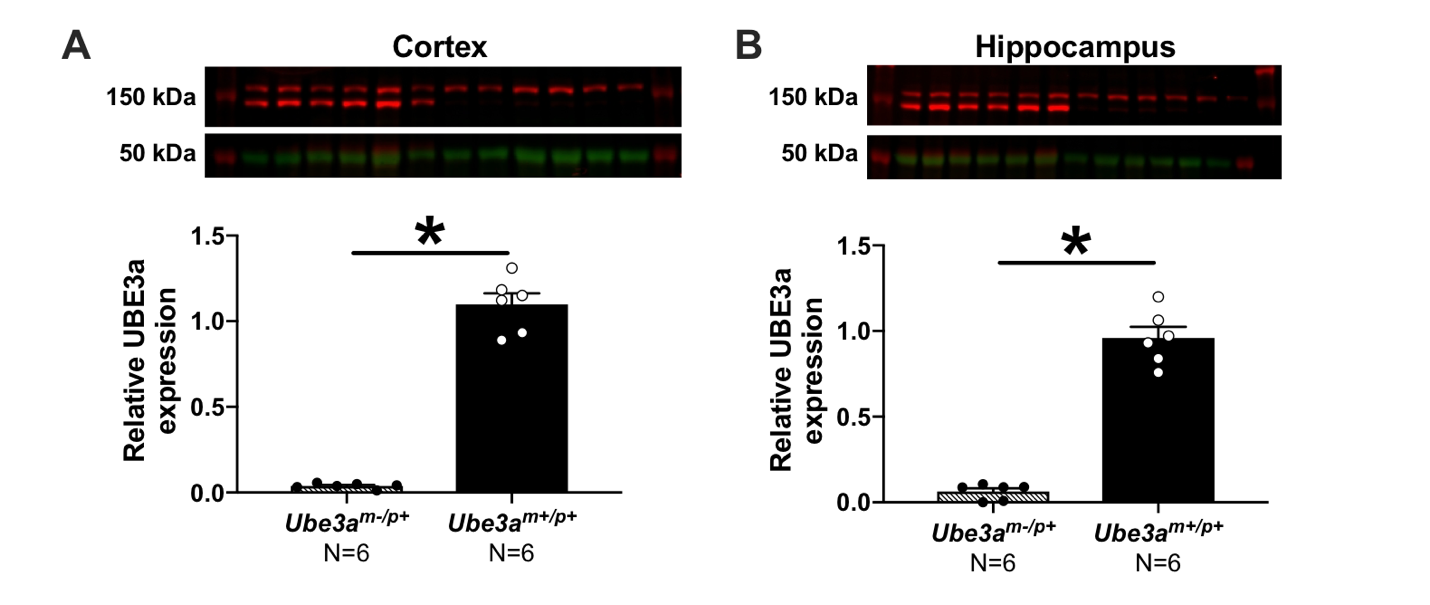


**Supplementary Figure S2. Reduced UBE3a protein in *Ube3a^m-/p+^* rats.** Western blots of brain samples from *Ube3a^m-/p+^* and *Ube3a^m+/p+^* rats show a lack of UBE3a expression (around 95 kDa; red) compared to beta-Tubulin expression (around 50 kDa; green). The upper band was approximately >100 kDa and the lower band was <100 kDa. Quantification confirmed that *Ube3a^m-/p+^* rats had significantly lower relative expression of UBE3a in both the A) cortex and B) hippocampus compared to wildtypes. Analyses include both males and females. **p* < 0.05, Student’s *t*-test. Quantitation was assessed on the lower band.


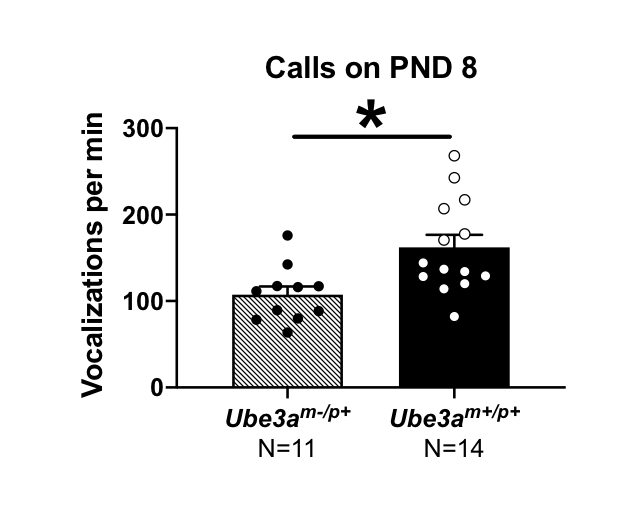


**Supplementary Figure S3. Reduced rate of isolation-induced pup ultrasonic vocalizations corroborated in an independent lab.** A research team at Baylor College of Medicine also discovered *Ube3a^m-/p+^* pups to make significantly fewer calls than wildtype littermates in an independent cohort, illustrating replication. Analyses include both males and females. **p* < 0.05, Student’s *t-*test.

**Supplementary Figure S4. Intact novel object recognition in *Ube3a^m-/p+^* rats.** A) Utilizing automated tracking software, both *Ube3a^m-/p+^* and *Ube3a^m+/p+^* rats were found to spend significantly more time investigating the novel object than the familiar object. B) The same result was found when sniff time was hand-scored by a trained observer blind to genotype. Analyses include both males and females. **p* < 0.05, paired *t*-test.
